# Test-takers’ perspectives on consumer genetic testing for hereditary cancer risk

**DOI:** 10.3389/fgene.2024.1374602

**Published:** 2024-07-10

**Authors:** Madison K. Kilbride, Lisa Jay Kessler, Brigitte Cronier, Jacqueline J. Park, Cara N. Cacioppo, Jordyn Beem, Angela R. Bradbury

**Affiliations:** ^1^ Department of Philosophy, University of Utah, Salt Lake City, UT, United States; ^2^ Master of Science Program in Genetic Counseling, Perelman School of Medicine, University of Pennsylvania, Philadelphia, PA, United States; ^3^ Kahlert School of Computing, University of Utah, Salt Lake City, UT, United States; ^4^ Penn Telegenetics Program, University of Pennsylvania, Philadelphia, PA, United States; ^5^ University of Louisville, Louisville, KY, United States; ^6^ Division of Hematology-Oncology, Abramson Cancer Center, University of Pennsylvania, Philadelphia, PA, United States; ^7^ Department of Medical Ethics and Health Policy, University of Pennsylvania, Philadelphia, PA, United States

**Keywords:** consumer genetic testing, cancer risk, participant experiences, behavioral changes, interview

## Abstract

**Purpose:**

With few exceptions, research on consumer genetic testing for hereditary cancer risk has focused on tests with limited predictive value and clinical utility. Our study advances the existing literature by exploring the experiences and behaviors of individuals who have taken modern consumer genetic tests for cancer susceptibility that, unlike earlier tests, screen for medically significant variants.

**Methods:**

We interviewed 30 individuals who had undergone consumer genetic testing for hereditary cancer risk between 2014 and 2019. We explored participants’ pre-test sentiments (7 items), experiences receiving results (5 items), behavioral and health-related changes (6 items), and attitudes and beliefs (3 items). Data were analyzed for thematic content.

**Results:**

Most participants reported a personal (n = 6) and/or family history (n = 24) of cancer, which influenced their choice to pursue testing. Before testing, most participants did not consult with a physician (n = 25) or receive genetic counseling (n = 23). Nevertheless, the majority felt that they understood test-related information (n = 20) and their results (n = 20), though a considerable number reported experiencing negative emotions related to their results. Most also shared their results with family members (n = 27). Overall, participants’ attitudes towards consumer genetic testing for cancer risk were predominantly positive (n = 23).

**Conclusion:**

This study offers new insights into how individuals use and perceive modern consumer genetic tests for hereditary cancer risk, focusing on their perceptions of the risks, benefits, and limitations of these services. Understanding test-takers’ perspectives can potentially inform improvements aimed at ensuring that tests meet users’ needs and deliver clinically valuable genetic risk assessments.

## Introduction

Genetic tests for the evaluation of hereditary cancer risk are increasingly accessible to consumers outside the clinical setting. Today’s consumer genetic tests can be broadly categorized into two types. The first type includes tests ordered directly by consumers without the involvement of healthcare providers. These testing services do not offer pre- or post-test genetic counseling, and results are delivered to the consumer through an online portal without guidance from a healthcare provider. Among companies offering this type of testing, only 23 and Me has been authorized by the US Food and Drug Administration (FDA) to market genetic tests for hereditary cancer risk. (23andMe, 2024). However, these tests are not considered clinical-grade and require confirmation before they can be used to make medical decisions about risk management.

The second type of consumer genetic test requires a physician’s order, even though testing is initiated by the consumer. Often, the physician is part of an independent network contracted by the genetic testing company to provide specific services. While pre-test genetic counseling is typically not included, companies offering this type of test usually provide post-test counseling as part of their service, although counseling may be limited to individuals receiving pathogenic or likely pathogenic results. Prominent companies in this space, such as Color Genomics and Invitae, offer clinical-grade tests that cover the same genes and utilize the same sequencing technology as tests routinely ordered in clinical settings. (Color Health, 2024; Invitae Corporation, 2024).

Scholars, private companies, and other stakeholders use different terms to describe these two broad types of consumer genetic testing services. (Kilbride and Bradbury, 2020). Tests in the first category (e.g., from 23andMe) are widely known as “direct-to-consumer genetic tests/testing” (DTC-GT) because consumers can order them without involving a physician. (Houriya Ayoubieh et al., 2023). In the United States, DTC-GT for monogenic hereditary cancer risks have been available since 2018 when 23andMe received FDA authorization to offer a limited *BRCA1/BRCA2* test as part of its service. The company has since expanded its offering to include 44 *BRCA1/BRCA2* variants and two variants for *MUTYH*-Associated Polyposis. (Food and Drug Administration, 2022; 23andMe, 2024). Tests in the second category (e.g., from Color and Invitae) are often referred to as “provider-mediated genetic tests/testing” (PM-GT), emphasizing that physicians, rather than consumers, are responsible for ordering them. (Majumder et al., 2021). In the United States, PM-GT for monogenic hereditary cancer risks have been available since at least 2015. (Cutler, 2024).

Despite differing with respect to who orders the tests—consumers for DTC-GT and physicians for PM-GT—these two models share several similarities. (Swetlitz, 2022). First, both types of tests increasingly target similar genes and conditions, including those related to cancer predisposition. Although PM-GT currently offers more comprehensive services, both models are trending towards more extensive offerings. Second, both types of tests are easily accessible, typically through company websites. This contrasts with clinic-based testing, which is often only available to individuals with a specific medical indication, such as a family history of cancer. (Hampel et al., 2015; Owens et al., 2019). Third, both types of tests lack adequate consumer support, with neither providing pre-test genetic counseling and PM-GT offering only limited post-test counseling. These practices diverge from professional recommendations that stress the importance of pre-test counseling to ensure informed consent and post-test counseling to help test-takers understand their results, whether positive, negative, or variant of uncertain significance (VUS). (Robson et al., 2015; Cancer Genetics Editorial Board, 2023).

Given the similarities between DTC-GT and PM-GT, we classify both under the broad category of consumer genetic testing. Throughout this paper, we will refer to tests from both models as “consumer genetic tests” to capture the commonalities between the two approaches.

With few exceptions, most of the research on consumer genetic testing for hereditary cancer risk has focused on traditional DTC-GT offerings that provide risk estimates based on low-penetrance variants, an approach known as single-nucleotide polymorphism (SNP) profiling or single-nucleotide variation profiling. (Bloss et al., 2011; Dohany et al., 2012; Sturm and Manickam, 2012; Bloss et al., 2013; Francke et al., 2013; Roberts and Ostergren, 2013; Carere et al., 2015; Gray et al., 2017; Roberts et al., 2017). Compared to many consumer tests that are currently available on the market and report on high- and moderate-penetrance single-gene variants, SNP profiling typically has low predictive value for future disease risk and limited clinical utility. (Liu et al., 2021; Sud et al., 2021). Overall, research has not found strong evidence of adverse outcomes for individuals who have undergone SNP-based DTC-GT, nor does it indicate that people make significant health- or lifestyle-related changes following such testing. (Bloss et al., 2011; Bloss et al., 2013; Carere et al., 2015; Gray et al., 2017; Roberts et al., 2017).

Given the differences between earlier consumer genetic tests and those that are now available, it remains uncertain whether the experiences and behaviors of today’s test-takers mirror those of the past. To address this gap in the existing literature, we conducted semi-structured interviews with individuals who took a consumer genetic test between 2014 and 2019 and received either a positive result or a VUS from 23andMe, Color Genomics, or Promethease, a third-party company that analyzes raw data from genetic tests. Our research sought to explore the experiences and behaviors of individuals who have undergone consumer genetic testing for hereditary cancer risk in the modern era, with the aim of uncovering new insights into how test-takers use and perceive these tests and to inform future prospective and quantitative research.

## Methods

### Participants

This study was approved by the University of Pennsylvania Institutional Review Board. Eligible participants included adults who had undergone consumer genetic testing—either DTC-GT or PM-GT—for hereditary cancer risk between 2014 and 2019 and received a positive result or a VUS. Participant eligibility was determined based on information from their medical chart or through self-report. We selected 2014 as the starting point because it marked the beginning of the 5-year period preceding our interviews. This timeframe allowed us to capture relatively recent test-taker experiences while still providing a sufficient window for recruiting participants. Other than the ability to communicate in English and by phone, there were no other inclusion or exclusion criteria.

### Recruitment strategy

Participants were recruited using a variety of approaches. We posted information about the study on the Basser Center’s website, a center at Penn Medicine that is focused on the research, treatment, and prevention of BRCA-related cancers. We also identified and contacted individuals through Penn’s Cancer Risk Evaluation Program (CREP) database and the Prospective Registry of Multiplex Testing (PROMPT). Additionally, Penn Medicine healthcare providers identified and referred potential participants to the study team. Participants recruited through Penn had sought clinical follow-up at the institution after receiving a positive result or a VUS from a consumer genetic test. Lastly, we asked participants to refer their eligible first-degree relatives to the study team (i.e., snowball sampling). Interested individuals were given a written copy of the Informed Consent and HIPAA Authorization Form and asked to provide verbal consent at the interview. Each participant received a $20 gift card in recognition of their contribution.

### Interview guide

A semi-structured interview guide was developed to explore participants’ experiences undergoing consumer genetic testing. Open-ended questions explored participants’ pre-test sentiments and experiences (7 items), experiences receiving test results (5 items), behavioral and health-related changes following receipt of results (6 items), and attitudes and beliefs about consumer genetic testing for hereditary cancer risk (3 items). Interviews were conducted between July 2020 and March 2021 by phone, lasted approximately 45 min to 1 hour, and were audio recorded and transcribed.

### Qualitative analyses

We used a modified grounded theory approach to identify emerging themes from individual items (e.g., constructs) in our interviews. (Kennedy and Lingard, 2006; Tavakol et al., 2006). The principal investigator and at least one additional member of the study team collaboratively reviewed the initial responses to open-ended questions from a sub-sample of participants to identify emerging primary and secondary themes and develop a coding schema. As new themes emerged in subsequent interviews, the coding schema was refined accordingly. Once the coding schema was established, two study team members independently coded the open-ended responses from the remaining transcripts. In the event of strong inter-coder disagreement, the two coders, along with a third member of the study team, discussed and resolved discrepancies. To ensure reliability, inter-coder agreement was measured with the kappa statistic. A kappa coefficient (Κ) of 0.8 was the cutoff for adequate agreement among coders. Frequency counts for the full range of primary and secondary themes are included to inform future prospective and quantitative research. Responses could be coded for more than one theme.

## Results

Participant Characteristics: Participants were a mean age of 45.2 and predominantly female (see Table 1). A significant majority of participants (n = 21) used a genetic test from Color Genomics. The remaining participants (n = 9) used a genetic test from 23andMe, with three also utilizing a third-party raw data analysis company (Promethease). Most participants received a positive result (n = 24) from their genetic test, while a small number received either a positive result and a VUS (n = 2) or only a VUS (n = 3). Three individuals initially took a genetic test from 23andMe, which yielded negative results. However, after submitting their raw data to Promethease, they received positive results. All three of these positive results from Promethease were later determined to be false positives, as clinical confirmation testing concluded that the individuals did not carry the reported genetic variants.

**TABLE 1:** Participant Characteristics (N = 30).

Characteristics	Value
Age (mean, range)	45.2 (25–79)
Sex (N) (%)	
Female	22 (73.3%)
Male	8 (26.7%)
Education (N) (%)	
Education consistent with college degree/college degree or higher	24 (80%)
Unknown or less than a college degree	6 (20%)
Consumer genetic testing company (N) (%)	
Color Genomics	21 (70%)
23andMe	9 (30%)
23andMe with submission of raw data to Promethease	3 (10%)
Results (N) (%)	
Positive	24 (80%)
Positive and VUS	2 (6.7%)
VUS only	3 (10%)
False positive (confirmed negative through clinical testing)	3 (10%)
Genes (positive results) (N) (%)	
*BRCA1/BRCA2*	14 (46.7%)
*CHEK2*	1 (3.3%)
*ATM*	6 (20%)
*BRIP1*	1 (3.3%)
Family history of cancer (N) (%)	
Yes	24 (80%)
No	4 (13.3%)
Unknown	2 (6.7%)
Personal history of cancer (N) (%)	
Yes	6 (20%)
No	24 (80%)

## Pre-test sentiments and experiences

### Motivations for testing

Participants were invited to discuss their motivations for pursuing consumer genetic testing. A common theme was the presence of a known cancer susceptibility mutation within their family (n = 8) or a family history of cancer (n = 4). However, some participants pursued testing because their family history is unknown (n = 4) (see Table 2).

**TABLE 2:** Reasons for pursuing consumer genetic testing for cancer risk (N = 30).

Why were you interested in consumer genetic testing?	Frequency counts for each coded theme
Mutation in family or personal/family history of cancer	
Known mutation in family	8
Family history of cancer	4
Personal history of cancer	2
Information	
Knowledge is power	5
Curiosity	5
Recreation	2
Cost	
Test was discounted, a gift, or free	6
Affordability	5
Information for relatives or about ancestry	
To gain information for relatives	4
Unknown history in relatives	4
Interested in ancestry or finding biological relatives	2
Adopted and do not know family history	1
Alternative to medical testing	
Convenience	5
Privacy considerations (e.g., consumer genetic testing felt more confidential)	2
Medical information or reproductive planning	
To understand ongoing medical issues or guide current cancer treatment	3
Reproductive or family planning	2
Altruistic reasons or to contribute to genetics research	2

“Yeah, so…my dad’s side has a very, very strong family history of cancer. And no man in his family had lived beyond age 62. My dad’s the first one to live past the age of 62…so I had a strong suspicion that there was something in the family and around the age of 30 is where it would start mattering to me as a woman of reproductive age because if it was a *BRCA* gene I knew that I would have to start my breast cancer screening.”

∼36-year-old female, *BRIP1* VUS, Color Genomics

Other common reasons for testing included the test being discounted or free (n = 6); the belief that knowledge is power (n = 5); curiosity (n = 5); convenience (n = 5); affordability (n = 5); and the desire to acquire information for relatives (n = 4) (see Table 2).

Regarding the ways in which participants became aware of the genetic test that they took, some reported that they learned about it through an offering at their workplace (n = 9).

“It was offered through my employer. We were offered genetic testing. I did have the availability of free genetic testing. And I’m very grateful, because I do not think I would have pursued it otherwise. Honestly, if it was – if it could have been 50 bucks, I would have been like, “No, I’m all set.” So, I’m very grateful.”

∼34-year-old female, *BRCA1* positive, Color Genomics.

Other sources included television, social media, or email advertisements (n = 6); suggestions by another person (n = 6); discovery on their own (e.g., through an Internet search) (n = 5); receipt of the test as a gift (n = 4); or through testing by another family member (n = 4).

### Interactions and communication with healthcare professionals

Participants were asked about their interactions with healthcare professionals before undergoing genetic testing. The majority reported no conversations with a physician prior to testing (n = 25). A few discussed whether to pursue testing with their physician (n = 4), though some did not have a primary care physician at the time (n = 3). Most participants reported no experience with genetic counseling before testing (n = 23). A few had previously attended a relative’s genetic counseling session (n = 2), had worked with genetic counselors in a professional setting (n = 3), or had undergone prior genetic testing (n = 1).

Among those who took Color’s test (n = 21), most reported that their test was ordered by a company-provided physician (n = 13). A few maintained that they ordered their own test (n = 2) or that their own physician ordered it for them (n = 1). Several participants could not recall who ordered their test (n = 3).

### Recollection of the informed consent process

When asked about their experience with the informed consent process for genetic testing, half of participants expressed having very little recollection of the process (n = 15) or being unable to recall details (n = 3). Many recalled there being an online consent (n = 10), or consent forms included in the test kit (n = 3). Several participants reported that the content of the consent was lengthy (n = 5), though one individual remembered it being short (n = 1). Many participants reported that they did not read the content of the informed consent (n = 7).

“I believe it was all online, and I just like read something, I think, and like verified that I’ve read it…I definitely know that I had to do some sort of consent, obviously. But I do not, I would be lying if I said I remember too much about it.”

∼34-year-old female, *BRCA1* positive, Color Genomics.

While a few participants shared that they did not question the informed consent because it came from a trusted source (n = 2) or felt that it was clear (n = 1), others expressed feeling that they may have signed away rights (n = 1) or that they were not confident in the company’s protections (n = 1).

### Understanding of test-related information

We asked participants to share how well they felt they understood company-provided information about genetic testing, including information that was presented through a website or was part of the informed consent process. The majority reported that the company’s information was clear and understandable (n = 20), with several maintaining that the disclaimers were adequate (n = 2) or that they had a better understanding due to their medical background (n = 1).

“As far as the health information, I felt that I understood pretty well what they were saying. That, you know, the way they tell you have an increased risk or increased likelihood or that they detected a variant. They do have disclaimers on there and make sure you discuss the findings with a physician. I thought that the information was relatively straightforward.”

∼29-year-old male, *BRCA1* positive, 23andMe.

Some participants, however, reported limited understanding of testing or the testing process, expressing a range of negative sentiments. These included (each reported once): not understanding anything; not appreciating the severity of the risks associated with the genes tested; feeling shocked, unprepared, or overwhelmed when results were disclosed; not having a clear understanding of how consumer genetic tests differed from each other; feeling that the test was primarily marketed for ancestry or traits; being unclear about the timeline of the testing process; feeling that the test results disclosure process was not clearly explained upfront; being unclear about how testing would be performed; and being unclear about how comprehensive the test would be.

## Experiences receiving test results

### Test result disclosure

Participants were asked to recall how they received their genetic test results. Among those who used 23andMe’s test (n = 9), most received their results through a link sent to them by email or through a login portal (n = 8), with a few mentioning that they also received their results by mail (n = 2) or that they could not recall (n = 1). Among the participants who used Color’s test (n = 21), the majority remembered receiving their results during a phone conversation with a genetic counselor (n = 15).

“I got an email that my Color results are in and I had to schedule an appointment to review my results. So, I got an email from a board-certified genetic counselor saying that they’ve completed the analysis of my sample and health history, please schedule a time to review the results with me over the phone. So, you review your results over the phone. And then as you’re over the phone, you can also see them on the screen. But you cannot see them on the screen until you schedule an appointment.”

∼29-year-old female, *BRCA1* positive, Color Genomics.

### Understanding of results

Participants were asked to discuss the extent to which they understood their test results and to recall any aspects that they found confusing or unclear. The majority reported that they understood their results (n = 20).

“I understood. I thought it was really clear. It was just that your mind is racing when they’re sharing this information with you. I think they sent me the pdf version of my whole report. That was really helpful because I was able to show that to other providers. I shared that with family members who needed to understand what I had heard. But it was something I could go back and review as I kind of processed and calmed down a little bit.”

∼48-year-old female, *BRCA2* positive, Color Genomics.

Some participants reported feeling that the information provided was confusing (n = 4) or contradictory (n = 1), wanting more information about a VUS or an “unknown variant” (n = 1), being unclear about the impact of results in terms of cancer risk (n = 1), feeling confused about whether the phrase “increased risk” in the results report meant that the test result was positive (n = 1), being unclear about what proactive steps to take (n = 2), and wanting more clarity about the penetrance of a mutation (n = 1).

“Um, I understood my results. The only thing that caused me slight anxiety was what did it mean. And they, they do not have a lot of information in general on this. Um, so I, um you know, I just wanted more information, but I felt like the counselor was very good.”

∼54-year-old female, *ATM* positive, Color Genomics.

Participants reported utilizing or drawing upon various resources to help them understand their results, including their own medical or educational background (n = 3), independently accessed research (n = 5), their own background knowledge about a known familial mutation (n = 1), and revisiting previously provided information (n = 1). When asked about the amount of information that testing companies provided about results, one participant reported experiencing information overload (n = 1), while others felt that the information was insufficient (n = 3) or that they simply needed time to process it (n = 1).

### Emotional and psychological responses to results

When asked to share their feelings upon learning their results, participants reported experiencing multiple emotions that included a range of negative, positive, and neutral reactions (see Table 3). Many described feeling sad or upset (n = 13), fearful or concerned for themselves (n = 11), surprised or shocked (n = 8), concerned for their family (n = 5), or overwhelmed (n = 4).

**TABLE 3:** Emotional and psychological responses to test results (N = 30).

How did you personally feel after learning your results?	Frequency counts for each coded theme
Negative reactions	
Sad or upset	13
Concerned or fearful for self (not otherwise specified)	11
Surprised or shocked	8
Concern for family	5
Overwhelmed	4
Disappointment	3
Guilt about possibility of passing risk to their children	1
Wish to have known information earlier	1
Positive reactions	
Empowered or glad to know information (not otherwise specified)	7
Relief	4
Neutral responses	
Neutral response (e.g., not upset)	3
Unsurprised	3
Cognitive responses	
Reaction changed over time	3
Desired more information	3
Confused	2
Awareness of own mortality	1

“Very overwhelmed. I still feel very overwhelmed sometimes about it…So, just the unknown of that, and even breast cancer and melanoma, it all becomes very overwhelming and it comes up at different times. And it felt like that is terrifying…And there’s just so many, it’s like a domino effect, of it’s not just this, you know you have this positive gene, now you have to look at the ramifications and what does it affect? And once you start going you know the next thing and the next thing, it all gets very overwhelming…So, a lot of worry came up with that.”

∼29-year-old female, *BRCA1* positive, Color Genomics.

Some participants articulated that they felt empowered and glad to have obtained information about their cancer risk (n = 7) or were relieved (n = 4). Others experienced a range of neutral emotional responses, as well as a range of cognitive responses (see Table 3).

“I think I just felt more powerful. I think just having that much more information about the specific genes that were analyzed and, you know, any potential risk factors, and even though the VUS, like, knowing the extent of what the unknown is was very helpful. So, yeah, I think it just helped me feel more armed with knowledge.”

∼36-year-old female, *APC* VUS, Color Genomics

## Behavioral and health-related changes after test results

### Sharing results with healthcare providers and relatives

Participants were asked to recall with whom, if anyone, they shared their genetic test results. Most participants reported sharing their results with their relatives (n = 27), predominantly with siblings (n = 13), parents (n = 7), and children (n = 5). Most shared their results with their spouse or a partner (n = 16). Many participants shared results with friends (n = 10) and healthcare providers (n = 13). A few shared with co-workers (n = 4).

Participants who shared results with relatives reported sharing them so that their family members could get genetic testing (n = 16). Some participants shared results to receive support from others (n = 6), to educate others (n = 5), because they believed it was important that certain individuals know that they had undergone testing (n = 5), or to develop a medical plan for managing cancer risk (n = 4).

### Health, lifestyle, and financial changes based on test results

Participants were asked to discuss their strategies for managing cancer risk given their test results. Most expressed an intention to undergo cancer screening, such as mammography or colonoscopy (n = 19). Several indicated that they were seeing a specialist (n = 6), having clinical visits or follow-up (n = 4), considering prophylactic mastectomy (n = 3), or considering prophylactic oophorectomy-salpingectomy (n = 3). Other changes are outlined in Table 4.

**TABLE 4:** Behavioral and health-related changes after test results* (N = 30).

At this point in time, what are your plans, if any, regarding cancer risk management?	Frequency counts for each coded theme
Cancer screening	19
Seeing a breast specialist or other specialist	6
Only clinical visits or follow-up at this time	4
Considered prophylactic mastectomy	3
Considered prophylactic oophorectomy-salpingectomy	3
Taking medication (e.g., chemoprevention)	2
Completed prophylactic mastectomy	1
Enrolling in a clinical trial	1
Staying up to date on literature pertaining to result	1

What health-related or lifestyle changes, if any, have you made or do you plan to make?	Frequency counts for each coded theme
Already has good diet and at a healthy weight	8
Already physically active	7
Considered dietary changes or improvements	7
Increased physical activity	5
Preventative surgery	4
Taking supplements	3
More proactive in personal healthcare (not otherwise specified)	3
None (not otherwise specified)	2
Changed doctors	1
Considered alcohol reduction	1
Losing weight	1
Advocating for cancer screening or genetic testing	1
Changing own professional practice/recommending genetic testing to patients (participant is a healthcare provider)	1

In what ways, if any, have your results affected your current or future reproductive plans?*	Frequency counts for each coded theme
None (not otherwise specified)	5
Feels sense of urgency regarding timing	4
Still young and not immediately relevant	3
Might consider pre-implantation genetic testing (PGT)	3
Have intentionally tried to not let it impact plans	1
Could be a factor in deciding not to have children	1

What changes, if any, have you made to any insurance policies, including changing a policy, getting a new policy, or dropping a policy?	Frequency counts for each coded theme
None	23
Added money to health savings account	3
Made changes to health insurance to cover cancer screening costs	2
Took out life insurance	1
Took out life insurance on a child	1
Took out disability insurance	1
Considered changes but did not ultimately make a change	1

* Multiple individual questions are included in this table. Participant responses could be coded for more than one theme.

**Reported for participants of reproductive age (18–45).

“Double mastectomy,. I’ve talked to the doctors, I’ve increased all of the screenings…and I talked to my GYN, we’ve adjusted my hormones…and then increased supplements and stuff that are supposed to decrease risks of melanoma, and breast or uterine cancer and pancreatic cancer.”

∼46-year-old female, *BRCA2* positive, 23andMe

We asked about any health- or lifestyle-related changes that participants had made or were planning to make after receiving their results. Many reported that they were already maintaining a healthy diet and weight (n = 8) or were engaging in regular physical activity (n = 7). Others expressed an intention to modify behaviors related to diet (n = 7), exercise (n = 5), and preventive surgery (n = 4), among others. (Table 4).

Participants were also asked how their test results have influenced their current or future reproductive plans. Participants of reproductive age varied in their responses: some reported no change in their plans (n = 5), while others expressed a newfound sense of urgency regarding timing (n = 4) or an interest in pre-implantation genetic testing (PGT) (n = 3). Other impacts are outlined in Table 4.

When asked if they had made or planned to make modifications to their insurance policies after receiving their results, the majority reported that they had made no such changes (n = 23). (Table 4).

## Attitudes and beliefs about consumer genetic testing for hereditary cancer risk

### Attitudes toward consumer genetic testing

We asked participants several questions about their attitudes and beliefs concerning consumer genetic testing for hereditary cancer risk. When questioned about their personal sentiments toward these services, most expressed a positive attitude (n = 23).

“I think it’s awesome. I hope that there’s more direct-to-consumer testing for everything because it makes it more affordable for people who do not have healthcare, um proper health coverage.”

∼46-year-old female, *BRCA2* positive, 23andMe.

A few participants conveyed ambivalence (n= 5), acknowledging both the positive and negative aspects of these tests. Additionally, several participants believed that these tests are not appropriate for everyone but can be good for certain individuals (n = 8).

“If it is something that directly affected me, and that would affect my siblings, or my, you know, grandkids and stuff like that, and if it were recommended by a doctor, I would do it. But generally, I guess I do not believe people should just call up somebody and have their genetic makeup put together for no particular reason. …Look, it’s not for everybody unless there’s a medical need to do that.”

∼79-year-old male, *BRCA2* positive, Color Genomics

### Recommendations for improving consumer genetic tests

We were also interested in participants’ opinions on how consumer genetic testing for hereditary cancer risk could be improved, if at all. Our findings did not reveal a dominant suggestion. A few participants believed that tests could be improved by providing test-takers with more resources (e.g., genetic counseling) (n = 6), providing more support from healthcare providers (n = 4), and being more affordable (n = 4).

“I think, maybe if, I think if, if my results, um you know, if I’d gone through my results with some sort of professional or someone who could have sort of explained things with, along with, it as we went through would be helpful, you know, I do not know if that’s cost effective, but I think that would be a nice change to the whole process.”

∼35-year-old male, *BRCA2* positive, 23andMe.

### Potential disadvantages and advantages of consumer genetic testing

Lastly, participants were prompted to explore what they take to be the potential disadvantages and advantages of consumer genetic testing for cancer risk. The most cited disadvantage was the possibility of emotional distress (n = 15).

“The disadvantages are you find out you have a risk there’s nothing you can do about, or you find out there’s a risk and your insurance does not cover you to do what you need to do. Or, you find out you have a higher risk, and you do not have access to providers who can really help you sift through it. There’s a mental health aspect of it.”

∼ 48-year-old female, *BRCA2* positive, Color Genomics.

Among the disadvantages reported, some participants cited the potential for misunderstanding results (n = 6), not following up and pursuing appropriate medical action (n = 6), and not appreciating the limitations of genetic tests (n = 4). A range of other reported disadvantages are outlined in Table 5.

**TABLE 5:** Attitudes and beliefs about consumer genetic testing for cancer risk (N = 30).

What do you think are the potential harms or disadvantages of consumer genetic testing for cancer risk? What do you think are the potential benefits or advantages?	
Potential harms or disadvantages of consumer genetic testing	Frequency counts for each coded theme
Emotional stress	15
Potential to misunderstand results and take inappropriate medical actions based on them	6
Potential to receive results and not follow up with a healthcare provider	6
Limitations of genetic testing need to be understood	4
Potential to exacerbate health disparities	3
Absence of genetic counseling or guidance from a healthcare professional	3
Potential for results to lead to discrimination in general	3
Potential for false reassurance (e.g., feeling relieved by negative result when only limited testing has been completed)	3
Unexpected results, including non-paternity	3
Confusion involving a false positive	2
Insurance implications or financial toxicity	2
Responsibility is on the consumer to learn updates regarding variants of uncertain significance (VUS) and results over time	2
Uncertainty regarding results or results having no clear medical follow-up	2
Variability in quality of labs/companies in the consumer genetic testing space or lack of oversight	2
—	—

Potential benefits or advantages of consumer genetic testing	Frequency counts for each coded theme
Opportunity to make medical and/or lifestyle changes	15
Sense of control over health outcomes, “knowledge is power”	11
Beneficial to pursue outside medical establishment	7
--Beneficial (not otherwise specified)	3
--Reduces barriers to testing (e.g., making an appointment)	5
--Allows for privacy/autonomy	1
Better cancer screening	5
Potential to catch cancer/disease at an earlier stage due to knowledge of genetic risk or increased screening	4
Ability to gain knowledge about personal health (in general)	4
Less costly	3
Makes healthcare more efficient	2
Opportunity to get connected to research studies	2
Allows for more options for individuals to pursue genetic testing	1

The most frequently reported advantages were the opportunity to implement medical and/or lifestyle changes based on results (n = 15) and a sense of empowerment (n = 11). Some participants also noted that the consumer genetic testing route enables testing outside of the medical establishment (n = 7). Table 5.

“Um, and I would say the biggest benefits, yeah, I think I’m very optimistic about humans’ capabilities to learn things and take control of their own health when they're given the opportunity. So I think it’s a really good thing if people are encouraged to learn more about their own health across the board, even at the deepest levels.So yeah, I think biggest benefits are knowledge and, you know, what the person could do with it.”

∼36-year-old female, *APC* VUS, Color Genomics.

## Discussion

Previous research on consumer genetic testing for cancer risk has focused on tests with limited predictive value and clinical utility. (Bloss et al., 2011; Sturm and Manickam, 2012; Bloss et al., 2013; Roberts and Ostergren, 2013; Carere et al., 2015; Gray et al., 2017; Roberts et al., 2017; Liu et al., 2021; Sud et al., 2021). Our research updates and expands upon the existing literature by exploring the experiences of individuals who have taken consumer genetic tests for cancer susceptibility in the modern era. The current generation of tests differ from earlier ones by focusing on high- and moderate-penetrance gene variants, many of which have significant medical implications.

An important finding from our study was that many participants indicated that their decision to pursue testing was influenced by their personal or family history of cancer. Additionally, most did not seek advice from a physician or receive genetic counseling prior to testing. While the majority of participants felt that they understood both the company’s test-related information and their test results, a significant number reported experiencing negative emotions related to their results. Most also chose to share their test results with family members, often with the intention of prompting them to get tested. Overall, attitudes towards consumer genetic testing for cancer risk were predominantly positive, suggesting a high level of satisfaction and acceptance of these services.

The finding that personal or family history of cancer was a major factor influencing most participants’ decision to pursue genetic testing aligns with previous research. Studies have consistently demonstrated that, in both clinical and consumer settings, individuals with a personal or family history of cancer are more likely to pursue testing, often motivated by their elevated risk profiles. (Baer et al., 2010; Roberts et al., 2011; Su et al., 2011; Finney et al., 2012; Hall et al., 2012). However, this observation raises questions about why these individuals, many of whom had a medical indication for clinical testing, chose to pursue consumer genetic testing over clinic-based options. One potential explanation, alluded to by some of our participants, is the growing availability of genetic testing through workplace wellness programs, which could steer employees toward selecting the most convenient and readily accessible option. This idea is further supported by those participants who felt that pursuing testing outside traditional medical channels was beneficial, noting that it reduced barriers to testing, such as the need to make appointments, and allowed for greater privacy and autonomy during the testing process.

Participants’ choices and preferences for how they engage with healthcare providers before testing underscore their interest in using consumer genetic testing services for assessing hereditary cancer risk, as opposed to more traditional clinic-based approaches. Significantly, most participants did not directly consult or communicate with a physician—their own or one affiliated with a consumer genetic testing service—nor did they receive genetic counseling prior to testing. Even Color test-takers, who had the option to have their test ordered by their own physician, preferred to have it ordered by a company-provided physician instead. This pattern might reflect a broader preference to sidestep the traditional clinical route, possibly motivated by a desire to avoid the inconvenience associated with scheduling and attending multiple appointments, as some participants expressed. Given that participants were predominantly well-educated and likely had access to healthcare, other test-taker populations might choose consumer genetic testing options for different reasons. For instance, factors such as the perceived higher cost of clinical testing due to inadequate insurance or a lack of access to local genetics services—both recognized barriers in the literature—could influence their decisions. (Dusic et al., 2022). Exploring motivations for pursuing consumer genetic testing among diverse populations is an important avenue for future research.

Another notable finding from our study is that participants generally perceived the information provided by testing companies to be clear and understandable. While this might seem encouraging, the concern remains that companies may be oversimplifying the risks, benefits, and limitations of testing, or that participants may be overestimating how well they understand the information. Most participants also felt that they understood their results, with many finding the knowledge gained to be empowering. Still, a considerable number reported experiencing negative emotions related to their results, such as sadness or fear. Overall, previous research on consumer genetic testing for cancer risk has not reported significant distress among test-takers. (Bloss et al., 2011; Bloss et al., 2013). However, since these older tests were less medically significant than current offerings, outcomes from clinic-based cancer susceptibility testing may provide a better benchmark for comparison. To date, research on the psychological impact of receiving a positive result from genetic testing for hereditary cancer risk has yielded mixed findings. (van Oostrom et al., 2003; Hamilton et al., 2009; Bosch et al., 2012; Kastner et al., 2023). While some individuals may continue to experience distress, research based on clinical populations suggests that, over time, most individuals adapt well. (Hamilton et al., 2009; Hirschberg et al., 2015; Bradbury et al., 2016; Lumish et al., 2017; Hamilton and Robson, 2019; Bradbury et al., 2020). Since participants in the current study were recounting emotions and experiences from as far back as 6 years, their reports of initial distress after receiving their results may not predict adjustment challenges over the long term. Nevertheless, their distress raises concerns about the post-test support that consumer genetic testing companies offer. Future research is needed to assess the emotional and psychological effects of contemporary consumer genetic testing for cancer risk as compared to clinic-based testing.

Reassuringly, and consistent with patterns observed in research involving clinical populations, we found that the majority of participants in our study chose to share their genetic test results with relatives. (Montgomery et al., 2013; Hunter et al., 2023). Their main reason for sharing was to encourage their biological relatives to undertake their own testing for risk assessment. This suggests that important health information is being disseminated to relatives who may be at risk, even in the absence of genetic counseling before or after testing. Consequently, concerns about relatives not receiving crucial genetic information might not be a disadvantage of consumer genetic tests.

Our study revealed a notably positive attitude among the majority of participants towards consumer genetic testing for cancer risk, highlighting a robust interest in, and appetite for, these services. While this important finding underscores growing acceptance and enthusiasm for these services, it was still the case that a considerable number of participants had mixed feelings about these tests or doubted their suitability for all potential test-takers. Participants identified a range of disadvantages and advantages associated with consumer genetic testing. The most cited downside was the distress stemming from undergoing the testing process and receiving results. This disadvantage could potentially be mitigated by increasing pre- and post-test counseling, which could help individuals cope with their distress and understand their results. Conversely, the most discussed advantage of consumer genetic testing is that it enables individuals to make medical changes based on their results, especially with respect to managing cancer risk. However, this advantage is not unique or exclusive to consumer genetic tests; clinical tests offer similar benefits, typically with more comprehensive guidance from genetics providers who can ensure that test-takers receive appropriate follow-up care. Importantly, most participants reported that they are following cancer screening recommendations, with some considering potential surgical interventions in the future. This finding suggests that consumer genetic test-takers pursue similar follow-up care to clinical test-takers. (Burton et al., 2012; Metcalfe et al., 2019). Still, given our participants’ high health literacy and likely access to care, these results may not be representative of broader populations.

Our study has several limitations. First, our sample is relatively small, especially for participants who took 23andMe’s test. Second, the population in our study was predominantly female, highly educated, and had a family history of cancer. Moreover, nearly all participants had received positive or VUS results. Consequently, individuals from different backgrounds or those who received negative results may hold views that differ from our findings. Third, we interviewed participants at different times after they took their genetic test, with the longest interval being close to 6 years. As a result, our findings may not capture the evolution of sentiments over time. Additionally, due to this time lapse, participants might not recall their experiences as vividly as if they were interviewed shortly after taking the test. Fourth, our recruitment strategy may bias the results. By selecting participants who underwent consumer genetic testing and sought clinical follow-up at Penn, our study may over-represent individuals who are more proactive or knowledgeable about their health. This could lead to findings that are skewed towards more positive attitudes and outcomes associated with higher healthcare engagement, which may not accurately reflect the experiences of the broader population. Finally, it is important to acknowledge that some of the interview data in this study is based on consumer genetic tests that were available a decade ago. Genetic testing offerings have evolved over time, which could raise concerns about the relevance of the data to the current landscape of consumer genetic testing. However, with respect to cancer susceptibility genetic testing, the current test offered by Color remains largely unchanged since it first became available. While other companies have expanded their offerings—Invitae’s PM-GT service now covers 65 cancer susceptibility genes in its 167-gene panel, and 23andMe has expanded its Health and Ancestry service to screen for 44 variants in the *BRCA1* and *BRCA2* genes, compared to three previously, as well as conditions like *MUTYH*-associated polyposis—the general nature and scope of the tests remains similar to what our participants took. (Invitae Corporation, 2024; Me Inc, 2022). Therefore, while it is possible that today’s test-takers may have somewhat different attitudes towards the larger tests that are currently available, obsolescence of the data is not a major concern.

This study provides new insights into the experiences of individuals who have used consumer genetic testing to assess hereditary cancer risk in the modern era. The overall positive attitudes towards testing, as reflected in participants’ responses, suggests that there is strong consumer interest in these offerings. Nevertheless, our findings point to important areas for further investigation, such as whether additional pre- or post-test genetic counseling could better support individuals throughout the testing process and reduce negative emotions associated with results. By exploring test-takers’ perceptions of the risks, benefits, and limitations of consumer genetic testing services for cancer susceptibility, current and future research can inform improvements to these tests aimed at ensuring that they meet users’ needs and deliver clinically useful genetic risk assessments.

## Data Availability

The raw data supporting the conclusion of this article will be made available by the authors, without undue reservation.
